# Antioxidative Action of Ellagic Acid—A Kinetic DFT Study

**DOI:** 10.3390/antiox9070587

**Published:** 2020-07-06

**Authors:** Jelena Tošović, Urban Bren

**Affiliations:** 1 Faculty of Chemistry and Chemical Engineering, University of Maribor, Smetanova Street 17, SI-2000 Maribor, Slovenia; jelena.tosovic@um.si; 2 Department of Chemistry, Faculty of Science, University of Kragujevac, 12 Radoja Domanovića, 34000 Kragujevac, Serbia; 3 Faculty of Mathematics, Natural Sciences and Information Technologies, University of Primorska, Glagoljaška 8, SI-6000 Koper, Slovenia

**Keywords:** QM-ORSA, antioxidative mechanisms, reaction rate constants, physiological conditions, polyphenols

## Abstract

Although one can find numerous studies devoted to the investigation of antioxidative activity of ellagic acid (EA) in the scientific literature, the mechanisms of its action have not yet been fully clarified. Therefore, further kinetic studies are needed to understand its antioxidative capacity completely. This work aims to reveal the underlying molecular mechanisms responsible for the antioxidative action of EA. For this purpose, its reactions with HO^•^ and CCl_3_OO^•^ radicals were simulated at physiological conditions using the quantum mechanics-based test for overall free-radical scavenging activity. The density functional theory in combination with the conductor-like polarizable continuum solvation model was utilized. With HO^•^ radical EA conforms to the hydrogen atom transfer and radical adduct formation mechanisms, whereas sequential proton loss electron transfer mechanism is responsible for scavenging of CCl_3_OO^•^ radical. In addition, compared to trolox, EA was found more reactive toward HO^•^, but less reactive toward CCl_3_OO^•^. The calculated rate constants for the reactions of EA with both free radicals are in a very good agreement with the corresponding experimental values.

## 1. Introduction

In recent years, therapeutic applications of non-drug substances such as functional foods, are progressively increasing. Therefore, studies on functional foods represent a cutting-edge topic among nutritional scientists. The significance of bioactive compounds as functional supplements of foods has been well established due to their effectiveness in health promotion by disease prophylaxis or treatment. Special attention has been devoted to investigations of polyphenolic compounds, known for their various nutritional, biologic, and pharmacological effects. In order to provide the health benefits to the consumers, functional foods and nutraceuticals have been supplemented with these compounds in recent years [1].

**Figure 1:**
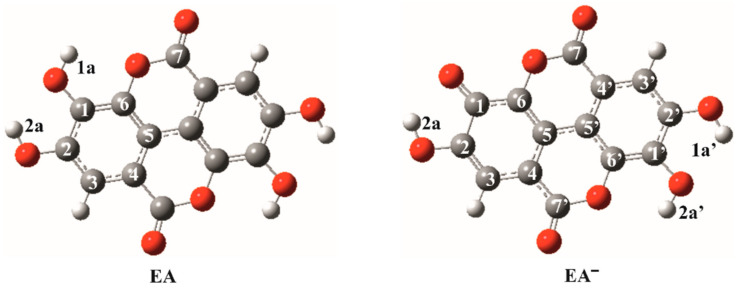
Optimized structures of neutral ellagic acid and its monoanion. Carbon atoms are depicted in gray, oxygen atoms in red, chlorine atoms in green and hydrogen atoms in white color. The atom labeling scheme and color coding are applied throughout the study.

Among polyphenolic compounds, ellagic acid (EA) attracts an ever-increasing interest due to its great potential in food technology, as well as in pharmaceutical, medical and cosmetic industries [2]. EA, a dimeric derivative of gallic acid, arises from acidic hydrolysis of ellagitannins. It represents a planar molecule which contains four hydroxyl and two lactone groups (see Figure 1). This dietary polyphenol can be found in a wide variety of fruits. Raspberries, cranberries, strawberries, grapes, as well as pomegranate seeds, are known for example for their high content of EA [3]. Other sources include pecans, walnuts and distilled beverages [4].

Like other dietary polyphenols, EA possesses a wide range of biological activities suggesting that it can exert strong beneficial effects on human health. In many epidemiological and experimental studies, anticarcinogenic, anti-inflammatory, antiviral, antibacterial, anti-atherosclerosis, antihypertensive, antihyperglycemic, cardioprotective and anti-fibrosis actions of EA have been demonstrated [5,6,7,8,9,10,11]. It can inhibit carcinogenesis by occupying sites (i.e., microsomal *P*-450 enzymes, glutathione-S -transferase or DNA), that would normally interact with ultimate carcinogens, through several mechanisms [12,13]. The anticarcinogenic effect of ellagic acid has been studied in various cancer cells. There it exhibits antiproliferative activity, combined with the ability to cause cell cycle arrest and to induce apoptosis [14]. The anticarcinogenic effects of ellagic acid have been observed in several cancer types: prostate, skin, esophageal and colon cancers [15]. Moreover, EA causes cell-specific responses, meaning that tumor cells are more susceptible to EA than normal cells [10]. In addition, EA prevents metabolic activation of aflatoxin B1, polycyclic aromatic hydrocarbons (PAHs) and nitroso compounds into ultimate carcinogens that cause DNA damage [12]. Due to its beneficial effects against a wide range of diseases, EA represents a great candidate for a therapeutic and chemopreventive agent, especially in the form of functional food supplements [16].

It has been shown that the high free radical scavenging activity of EA may be at least partially responsible for the observed in vivo biologic effects [14]. The presence of four hydroxyl groups enables EA to scavenge numerous reactive oxygen and nitrogen species and makes this compound a powerful antioxidant [17,18]. EA represents also a very efficient inhibitor of lipid peroxidation even at micromolar concentrations [19]. Furthermore, studies of Hassoun et al. showed that EA exhibits a better antioxidative efficacy against oxidative stress and lipid peroxidation than vitamin E [19]. Finally, besides numerous beneficial effects on human health, EA as a strong antioxidant can prolong shelf life and preserve the quality of foods [1].

In scientific literature, one can find a few theoretical studies devoted to the examination of the antioxidative activity of EA through thermodynamic and kinetic approaches. Marković et al. have shown that the thermochemical viability of different antioxidative mechanisms depends on the deprotonated portion of EA, the polarity of reaction media, as well as on the properties of the free radical [20]. Based on the calculated thermodynamic parameters, they have suggested that the hydrogen atom transfer (HAT) is the most favorable mechanism in nonpolar media, whereas sequential proton loss electron transfer (SPLET) is preferred in polar media, which is in agreement with results reported by Mazzone et al. [21]. Utilizing the transition state theory, Tiwari and Mishra have determined the rate constants for the reactions of EA (as well as its monomethyl and dimethyl derivatives) with hydroxyl (HO^•^), methoxy (CH_3_O^•^) and nitrogen dioxide (NO_2_^•^) radicals [22]. However, the calculated rate constants of HO^•^ and CH_3_O^•^ radicals have been overestimated by several orders of magnitude in comparison with the experimentally obtained values [17,18]. Galano et al. have also investigated several aspects related to the antioxidant activity of EA [17]. They have demonstrated that the free radical scavenging activity of EA does not decrease upon metabolism and provides continuous protection against oxidative stress. To the best of our knowledge, the results of Tiwari and Mishra and Galano et al. represent the only theoretical studies dedicated to kinetic investigations of antioxidative activity of EA [17,22].

However, one can find numerous experimental studies devoted to the examination of EA as an important component of various foods and beverages, its antioxidative mechanisms have not been fully clarified. Elucidation of the mechanisms by which dietary polyphenols prevent and suppress various diseases represents an important step in understanding their effects in vivo and may help the design of novel strategies for disease prophylaxis and treatment. Therefore, further kinetic investigations are needed to reveal and to fully understand the underlying molecular mechanisms responsible for the antioxidative action of EA. Consequently, the hydrogen atom transfer (HAT), radical adduct formation (RAF), sequential proton loss electron transfer (SPLET) and single electron transfer (SET) mechanisms [23,24,25] were studied by simulating the reactions of EA with two free radicals, HO^•^ and CCl_3_OO^•^, at physiological conditions (pH = 7.4 in aqueous solution) using quantum-chemical methods. An additional goal was to determine the relative antioxidative activity of EA, using trolox (6-hydroxy-2,5,7,8-tetramethylchroman-2-carboxylic acid, Tx) as a reference compound.

## 2. Materials and Methods

### 2.1. Computational Methods

All results were obtained from calculations using the density functional theory (DFT) approach. Full geometry optimizations and subsequent frequency calculations were performed using the hybrid meta M06-2X functional in conjunction with flexible 6-311++G(d,p) basis set and conductor-like polarizable continuum model (CPCM) [26], as implemented in the Gaussan 09, Revision D.01, software package [27]. Implicit water solution (dielectric constant, ε = 78.3553) was employed to mimic the physiological aqueous environment. The M06-2X functional was developed for studying main-group thermochemistry and kinetics [28,29]. In addition, this theoretical model has recently demonstrated robustness and very good overall performance in investigations of several related polyphenolic systems [30,31,32,33,34]. Restricted and unrestricted calculations were applied for the closed-shell and open-shell structures, respectively. The nature of the reactive species was confirmed by analyzing the results of the subsequent frequency calculations in the harmonic approximation: only real frequencies for equilibrium geometries and exactly one imaginary frequency for transition states (TSs) were obtained. The intrinsic reaction coordinate (IRC) calculations were additionally performed to verify each transition state. IRC represents the minimum energy reaction pathway (MERP) in mass-weighted cartesian coordinates between the transition state and the corresponding reactants and products. Moreover, the natural bond orbital (NBO) analysis was applied for all structures to obtain the corresponding partial atomic charges [35]. The IRC and NBO analyses were performed using default settings.

### 2.2. Quantum Mechanics-Based Test for Overall, Free-Radical Scavenging Activity

Four antioxidative mechanisms—HAT, RAF, SPLET and SET—were examined following the quantum mechanics-based test for overall free-radical scavenging activity (QM-ORSA) protocol [36], which was designed for studying free-radical reactions in solutions of different polarities. QM-ORSA represents a universal and quantitative method of evaluating the free radical scavenging activity of chemical compounds, that is, their primary antioxidant activity. This methodology involves revealing of all thermodynamically feasible reaction pathways included in the antioxidative process, which are subjected to subsequent kinetic investigations. By calculating the reaction pathways for all present acid–base forms of the investigated compound, QM-ORSA takes into account also the influence of pH. Namely, at a particular pH, the antioxidant can be present in different acid–base forms (cationic, neutral, monoanionic, dianionic, etc.) depending on its *pKa* values. The reliability of the QM-ORSA protocol was confirmed on the set of test reactions, where the correlation between the logarithms of the calculated and experimental rate constants was excellent (the R value is very close to one (0.99), the slope is very close to one (0.99), and the intercept is very close to zero (0.06) [36]. Moreover, the absolute error of the Gibbs activation free energies of 1.213 kJ mol^−1^ was significantly lower than the accepted computational accuracy of 4.184 kJ mol^−1^. Finally, this protocol has been successfully applied for several investigations of antioxidative activity in the scientific literature [31,32,34,37,38,39,40,41,42].

#### 2.2.1. Thermodynamic Considerations

The thermochemical viability of all possible reaction pathways and reaction sites included in the antioxidative process was investigated in terms of the reaction Gibbs free energies (Δ*G_r_*). The free energies of the examined reactions were determined at T = 298.15 K and P = 101,325 Pa. The exergonic (Δ*G_r_* < 0) and isoergonic (Δ*G_r_* ≈ 0) reaction paths were subjected to further kinetic calculations.

#### 2.2.2. Kinetic Considerations

Depending on the type of the mechanism, the reaction rate constants were obtained in two different ways. In the case of HAT and RAF mechanisms, where the transformation of reactants to products occurs over energy barriers, the Eckart method [43] also known as zero-tunneling method (ZCT-0) was applied. This method uses the Eckart function for generating the ground-state potential energy function based on information on the stationary points (reactants, transition state and products) along MERP. To perform the Eckart method calculations TheRate program [44] was utilized. For the electron transfer reactions involved in SPLET and SET mechanisms, the Marcus theory [45] was applied.

The overall reaction rate constants (*k*_overall_), which correspond to experimentally observed reaction rates of specific free-radical reactions, were calculated. The *k*_overall_ values were obtained as a sum over all acid–base species (*i*) present at the physiological pH (7.4) of the total reaction rate constant values (*k*_TOT_) multiplied by the corresponding molar fractions (*f*):

(1) $$k_{\mathrm{overall}}=\underset{\begin{array}{c} i=\{\mathrm{acid}-\mathrm{base}\\ \mathrm{species}\} \end{array}}{\sum }f\left(i\right)\times k_{\mathrm{TOT}}\left(i\right)$$

The *k*_TOT_ values for all acid–base species were obtained as sums of the reaction rate constants corresponding to each antioxidative mechanism (*j*):

(2) $$k_{\mathrm{TOT}}=\underset{\begin{array}{c} j=\{\mathrm{antioxidative}\\ \mathrm{mechanism}\} \end{array}}{\sum }k_{\mathrm{mech}}\left(j\right)$$

The *k*_mech_ is defined as a sum of reaction rate constants (*k*) belonging to the same antioxidative mechanism calculated at different reactive sites (*l*):

(3) $$k_{\mathrm{mech}}=\underset{\begin{array}{c} l=\{\mathrm{antioxidative}\;\\ \mathrm{pathway}\} \end{array}}{\sum }k\left(l\right)$$

The antioxidative pathway belongs to a specific antioxidative mechanism at a specific reactive site. To determine the relative contribution of an antioxidative pathway (*l*), the branching ratio, *Γ*(*l)*, was calculated using the following relation:

(4) $$\Gamma \left(l\right)=\frac{k\left(l\right)}{k_{\mathrm{overall}}}$$

#### 2.2.3. Relative Antioxidative Activity

The relative antioxidative activity of **EA** (*r*^T^) was calculated by dividing *k*_overall_ of EA with *k*_overall_ of **Tx**:

(5) $$r^{T}=\frac{k_{\mathrm{overall}}^{\mathrm{EA}}}{k_{\mathrm{overall}}^{\mathrm{Tx}}}$$

## 3. Results and Discussion

### 3.1. Thermodynamic Considerations

In the previous study of Galano at al. it was found that the dominant species of EA present at physiological conditions (pH = 7.4) are neutral (~10.7%) and monoanionic (EA^−^, ~89.3%) forms, which is also in accordance with the reported *pKa* values (Figure 1) [17].

To select favorable mechanistic pathways for further kinetic investigations of the antioxidative action of EA the Gibbs free energies of the following reactions of neutral species:

(6) $$\mathrm{HAT}:\;\mathrm{EA}+R^{•}\to {\mathrm{EA}}^{•}+\mathrm{RH}$$

(7) $$\mathrm{RAF}:\;\mathrm{EA}+R^{•}\to \;{\left[\mathrm{EA}-R\right]}^{•}$$

(8) $$\mathrm{SPLET}\;\left(I\;\mathrm{step}\right):\mathrm{EA}+{\mathrm{HO}}^{-}\to {\;\mathrm{EA}}^{-}+H_{2}O$$

(9) $$\mathrm{SPLET}\;\left(\mathrm{II}\;\mathrm{step}\right):{\;\mathrm{EA}}^{-}+R^{•}\to {\;\mathrm{EA}}^{•}+R^{-}$$

(10) $$\mathrm{SET}:\mathrm{EA}+R^{•}\to {\;\mathrm{EA}}^{•+}+R^{-}$$

as well as of monoanionic species:

(11) $$\mathrm{HAT}:{\;\mathrm{EA}}^{-}+R^{•}\to {\;\mathrm{EA}}^{•-}+\mathrm{RH}$$

(12) $$\mathrm{RAF}:{\;\mathrm{EA}}^{-}+R^{•}\to \;\;{\left[\mathrm{EA}-R\right]}^{•-}$$

(13) $$\mathrm{SPL}\mathrm{ET}\;\left(I\;\mathrm{step}\right):{\;\mathrm{EA}}^{-}+{\mathrm{HO}}^{-}\to {\;\mathrm{EA}}^{2-}+H_{2}O$$

(14) $$\mathrm{SPLET}\;\left(\mathrm{II}\;\mathrm{step}\right):{\;\mathrm{EA}}^{2-}+R^{•}\to {\;\mathrm{EA}}^{•-}+R^{-}$$

(15) $$\mathrm{SET}:{\;\mathrm{EA}}^{-}+R^{•}\to {\;\mathrm{EA}}^{•}+R^{-}$$

had to be examined first. In reactions (6)–(15) R^•^ stands for HO^•^ or CCl_3_OO^•^. The HO^•^ represents the most electrophilic among the oxygen-centered radicals capable of reacting immediately after its formation with almost any molecule in the vicinity. It is responsible for 60% to 70% of the tissue damage caused by ionizing radiations and most oxidative damage to DNA [23]. CCl_3_OO^•^ is generated in the organism during the metabolism of CCl_4_, a well-known liver toxin. As most of the oxygen radicals, CCl_3_OO^•^ reacts with various biomolecules such as proteins, DNA and lipids [46,47]. In addition, CCl_3_OO^•^ was specifically selected because it is often used in experimental studies to imitate larger peroxyl radicals [48]. A wide variety of experimental studies have been indeed conducted in order to elucidate an effective scavenger of this radical, especially among the naturally occurring antioxidants. However, to the best of our knowledge, computational investigations regarding the reactivity of CCl_3_OO^•^ remain surprisingly scarce.

Structures of EA and its monoanion employed in the present study are consistent with the structures published in previous papers [17,20]. The calculated reaction free energies are summarized in Table 1. In the case of the neutral species, only half of the positions in the molecule must be considered explicitly due to the symmetry, whereas in the case of the monoanion the symmetry is broken and all the sites must be considered explicitly.

According to the highly exergonic Δ*G_r_* values, EA and EA^−^ can scavenge both free radicals through HAT reaction pathways. In the case of EA, all four positions (1a = 1a’ and 2a = 2a’) are equally feasible, whereas in the case of EA^−^ the reaction pathway at position 2a’ becomes the most favorable. In the case of the RAF mechanism, the reaction pathways at positions 7 and 7′ are excluded from the examination. Namely, the significant partial positive charge of carbonyl carbons, makes these positions unsuitable for the attack of the studied electrophilic free radicals (Figure S1). All remaining reactive positions with HO^•^ radical are exergonic or isoergonic (Δ*G_r_* ≈ 0), suggesting that the RAF mechanism is thermodynamically favorable. As for CCl_3_OO^•^ radical, we were unable to locate the corresponding radical adduct for the reaction with EA^−^ at position 1 and all remaining positions are endergonic. These findings imply that RAF mechanism cannot be responsible for the antioxidative action of EA in the case of CCl_3_OO^•^ radical.

The basic environment provides conditions for proton loss from EA and EA^−^, to form EA^−^ and EA^2−^, respectively, which is reflected in the negative Δ*G_r_* values for the first step of the SPLET mechanism. Considering, that EA^2−^ represents the dominant form only at higher pH values (pH > 10), it is reasonable to assume that SPLET mechanism cannot be responsible for the antioxidative action of EA^−^ toward the studied selected free radicals [32,49]. On the other hand, the second step of the SPLET mechanism of EA deserves a careful inspection. Namely, electron transfer reaction is endergonic in the case of the highly reactive HO^•^, whereas it is isoergonic in the case of CCl_3_OO^•^ and should, therefore, be further examined. The higher reactivity of CCl_3_OO^•^ in comparison to HO^•^ during the electron transfer reaction can be explained by the strong negative inductive effect of the three chlorine atoms which increases the electron affinity of the radical. High Δ*G_r_* values for the SET reactions between EA and both studied free radicals indicate that this mechanism does not occur, whereas the SET reaction pathway of EA^−^ is identical to the second step of the SPLET mechanism of EA.

### 3.2. Kinetic Considerations

All exergonic and isoergonic reaction pathways were subjected to kinetic examination aimed at revealing the TSs and at calculating the corresponding activation free energies and reaction rate constants (Table 2).

**Figure 2:**
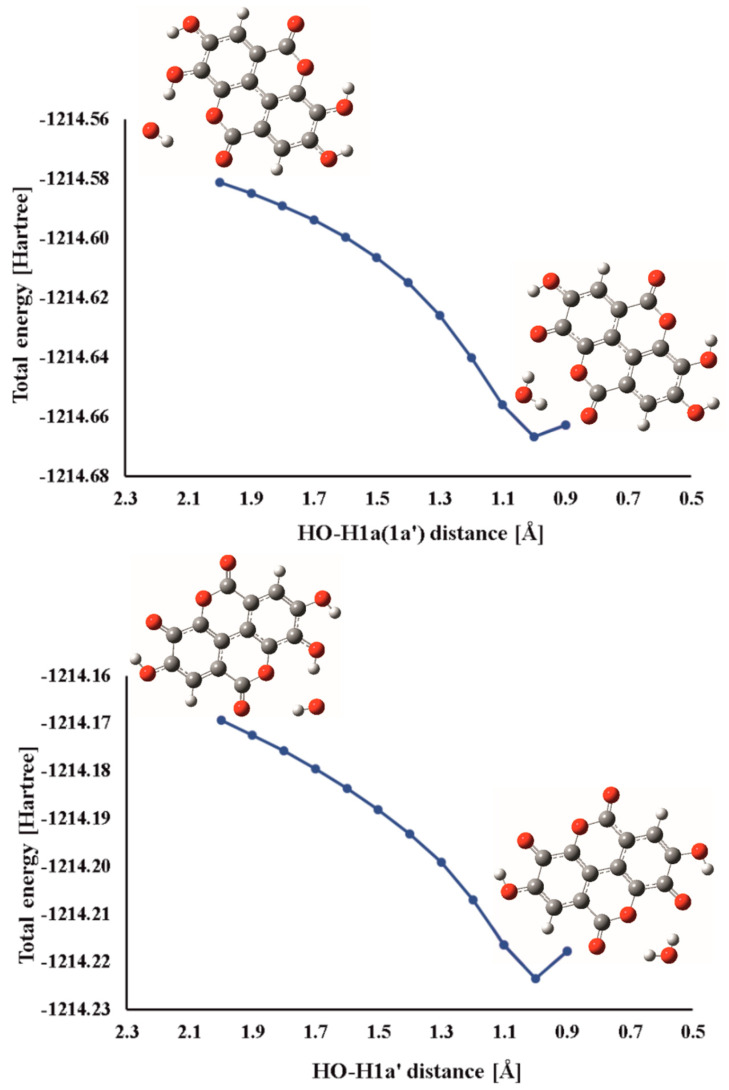
Dependence of total energy on the characteristic HO^•^–H distance during the hydrogen atom transfer between ellagic acid (top) or its monoanion (bottom) and HO^•^.

All our attempts to locate TSs for the HAT reactions of EA and EA^−^ with HO^•^ were unsuccessful, so it was reasonable to assume that all these processes are barrierless. To confirm this assumption, each reactive position was further investigated in the following manner. HO^•^ radical was positioned in the vicinity of the corresponding H atom and then allowed to approach the reactive center up to the formation of the products. Dependence of the total energy on the corresponding scan coordinate (HO^•^–H distance) was analyzed. Based on the monotonous decrease of total energy with decreasing of HO^•^–H distance it was concluded that these reactions are indeed barrierless and therefore diffusion-controlled, with the corresponding reaction rate constant of 1.91 × 10^9^ M^−1^ s^−1^. Two representative total energy profiles (one for the neutral form EA and one for the monoanion EA^−^) for HAT reactions are depicted in Figure 2.

**Figure 3:**
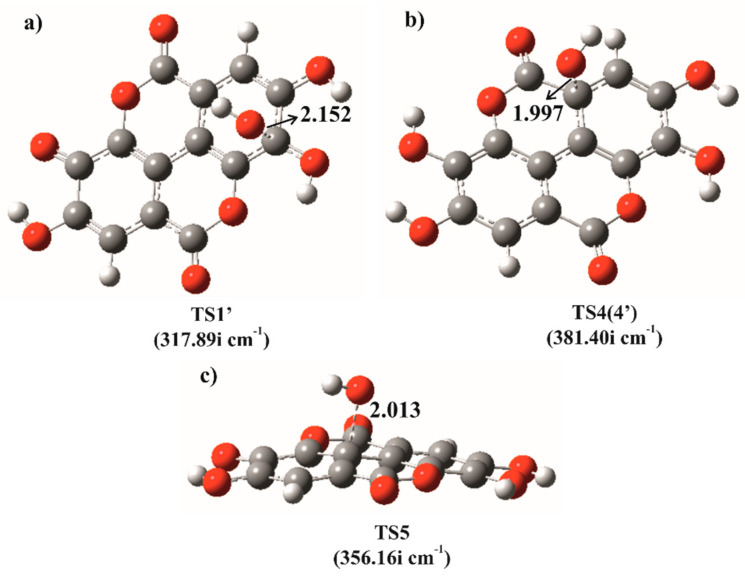
Representative examples of transition states obtained for the RAF reaction of EA and EA^−^ with HO^•^ at positions: (**a**) 1′ (EA^−^), (**b**) 4=4′ (EA) and (**c**) 5 (EA^−^). All distances are reported in Å.

A majority of TSs involved in the RAF mechanism of EA and EA^−^ with HO^•^ were successfully allocated (Cartesian coordinates of all TSs are provided in the Supplementary Materials). Two exceptions represent the reaction pathways at positions 2 and 6 of EA^−^, for which we were not able to locate TSs, despite numerous attempts. For this reason, the relaxed scan procedure applied for the HAT reaction pathways has also been employed in these two cases. The total energy of the system indeed monotonously decreases with decreasing HO^•^–C distance, so it can be concluded that these two processes are also diffusion-controlled (Figure S2). The increased affinity of C2 and C6 atoms toward HO^•^ in comparison to other positions is not surprising due to the strong mesomeric activating effect of O^−^ at ortho and para positions. The rate constants for the reactions at other positions are correspondingly reduced mostly by two orders of magnitude. The mutual characteristics of TSs obtained for the studied RAF reactions include relatively strong interactions of hydroxyl radical with π electrons of the aromatic ring, as well as the preserved planarity of the molecule (Figure 3a). Slower rates are observed for the reactions at positions 4 = 4′ of EA and 4′ of EA^−^, whereas the slowest reactions are those in positions 5 = 5′ of EA, as well as 5 and 5′ of EA^−^. In the first case, the π-interactions between the hydrogen of the hydroxyl radical and the aromatic ring is lost in TS (Figure 3b), whereas in the second, the reacting system becomes nonplanar and therefore less stable (Figure 3c). Additionally, the results of the IRC calculations for the representative TSs for the RAF mechanism with HO^•^ are shown in Figure S3.

**Figure 4:**
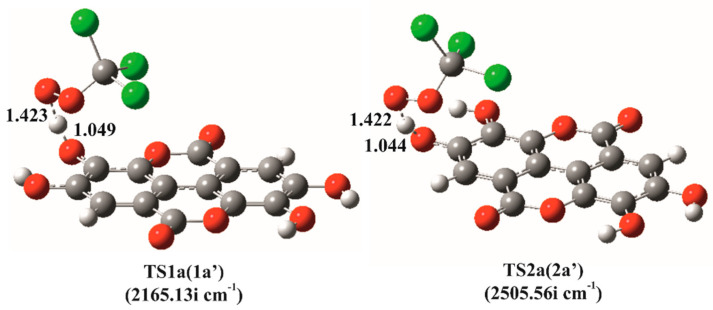
Optimized geometries of transition states for the hydrogen atom transfer (HAT) reaction pathways of ellagic acid with CCl_3_OO^•^ at positions 1a=1a’ and 2a=2a’. All distances are reported in Å.

For the HAT reactions between EA and CCl_3_OO^•^ both TSs were successfully located (Figure 4). The results of the IRC calculations are shown in Figure S4. In both TSs, the planarity of the system is preserved. As expected, the HAT reaction pathways with CCl_3_OO^•^ are slower than the corresponding reactions with HO^•^ (Table 2). On the other hand, we have encountered significant difficulties to locate TSs for the reactions of EA^−^ with CCl_3_OO^•^. Only one approximation of TS was revealed, using a similar procedure described in detail in a previous study of Tošović and Marković [32]. Namely, the energy profile of the reaction in 2a’ position is characterized by an extremely steep decrease to the energy minimum (Figure S5). It is worth pointing out that the corresponding energy maximum is characterized by a single desired strong imaginary vibrational frequency (1339.94i cm^−1^). Considering that the calculated $\Delta G_{a}^{‡}$ value is extremely high (~200 kJ mol^−1^) and the corresponding rate constant is tremendously small, the contribution of this reaction pathway to the overall antioxidative capacity of EA toward CCl_3_OO^•^ remains negligible. It is reasonable to assume that similar results would be observed in the case of the two remaining HAT reaction paths (at positions 2a and 1a’), so they were not considered further.

Figure S6 demonstrates a barrierless formation of EA^−^ in a proton loss reaction of the SPLET mechanism of EA, whereas the rate constant value of 1.56 × 10^9^ M^−1^ s^−1^ for the second step of the SPLET mechanism, i.e., the electron transfer reaction, indicates that this reaction is also practically diffusion controlled.

The obtained $k_{\mathrm{overall}}$ values amount to 9.70 × 10^9^ and 3.71 × 10^8^ M^−1^ s^−1^ for the reactions with HO^•^ and CCl_3_OO^•^ (Table 2), respectively and it is very interesting to compare them with the existing experimental results. In the study of Priyadarsini et al. [18], the rate constants for these reactions were determined in aqueous solution at pH=7 using pulse radiolysis technique and amount to 8.9 × 10^9^ and 1.4 × 10^8^ M^−1^ s^−1^ for the HO^•^ and CCl_3_OO^•^ radicals, respectively. Considering that the agreement between experimental and calculated reaction rate constants is very good, it can be concluded that the utilized computational approach successfully quantified reactivity of EA toward both studied free radicals.

To estimate the importance of each individual path to the overall antioxidative capacity of EA, the branching ratios were calculated (Table S1). The greatest *Γ* values in the case of HO^•^ belong to the diffusion-controlled reactions of monoanion, i.e., all HAT reaction pathways and two specific RAF reactions. On the other hand, the highest *Γ* values were obtained for the SPLET reaction paths between CCl_3_OO^•^ and the neutral form of EA.

Galano et al. reported the overall rate constant values for the reactions of EA with HO^•^ and CCl_3_OO^•^ radicals (among others) calculated solely based on the mechanisms in which the electron transfer reactions are involved, i.e., SPLET and SET, using a different theoretical model [17]. Our work suggests that in the case of CCl_3_OO^•^ radical the electron transfer reaction is indeed the predominant antioxidative pathway and the comparison of our results with the study of Galano et al. for overall rate gives a good agreement. On the other hand, our results indicate that SPLET and SET mechanisms are not favorable for scavenging of HO^•^ and no meaningful comparison with the work of Galano et al. can be made.

### 3.3. Relative Antioxidative Activity

According to the QM-ORSA protocol, a thermodynamic and kinetic study needs to also be performed for the reference compound, Tx, to determine relative antioxidative value, *r*^T^. The *k*_overall_ value for the reaction of Tx with HO^•^, calculated using an identical methodology and theoretical model under equal conditions, has been recently reported and amounts to 1.94 × 10^9^ M^−1^ s^−1^ [32]. On the other hand, to the best of our knowledge, the *k*_overall_ value for the reaction of Tx with Cl_3_COO^•^ is yet unknown. For this reason, all necessary calculations regarding this reaction had to be performed. The corresponding results and short discussion are provided in the Supplementary Materials. The obtained *k*_overall_ value for Tx reacting with Cl_3_COO^•^ is equal to 1.91 × 10^9^ M^−1^ s^−1^.

Based on the calculated *k*_overall_ values for the reactions of EA and Tx with HO^•^ and CCl_3_OO^•^ in aqueous solution the *r*^T^ values were determined. The obtained *r*^T^ values of 5.00 and 0.19 for the reactions with HO^•^ and CCl_3_OO^•^, respectively, imply that EA is more reactive toward HO^•^, but less reactive toward CCl_3_OO^•^ in comparison to Tx.

## 4. Conclusions

Antioxidants represent an important group of functional compounds that possess the ability to extend shelf life and maintain the quality of foods. More important, in biologic systems, antioxidants protect against oxidative stress and consequently help to prevent numerous diseases.

In this work, we investigated the antioxidative mechanisms of a dietary polyphenol EA by utilizing the QM-ORSA methodology. For this purpose, the reactions of EA with HO^•^ and CCl_3_OO^•^ radicals were simulated.

Highly exergonic Δ*G_r_* values indicate that EA and EA^−^ can scavenge both investigated free radicals through HAT reaction pathways. The RAF reaction pathways are thermodynamically possible in the case of the reactions with HO^•^, whereas the SPLET reaction mechanism is thermodynamically feasible in the case of CCl_3_OO^•^ radical. High Δ*G_r_* values for the SET reactions between EA and both studied free radicals indicate that this mechanism does not play a vital role.

Based on the obtained kinetic results, EA can scavenge HO^•^ primarily through HAT and RAF mechanisms, whereas SPLET mechanism is responsible for scavenging of the CCl_3_OO^•^ radical. Moreover, based on the calculated *r*^T^ values, EA is more reactive toward HO^•^, but less reactive toward CCl_3_OO^•^ than Tx.

Last but not least, the calculated overall reaction rate constants, *k*_overall_, for the reactions of EA with HO^•^ and CCl_3_OO^•^, respectively, are in a very good agreement with the experimental values, indicating that the applied computational methodology successfully quantified the reactivity of EA toward both investigated free radicals. Considering that antioxidative mechanisms in aqueous environments are extremely complex, the consensus between the calculated and available experimental data strongly supports the reaction mechanisms proposed in this work.

## Figures and Tables

**Table 1:** Gibbs energies Δ*G_r_* (kJ mol^−1^) of the reactions of ellagic acid (EA) and its monoanion (EA^−^) with HO^•^ and Cl_3_COO^•^; HAT, RAF, SPLET and SET denote hydrogen atom transfer, radical adduct formation, sequential proton loss electron transfer and single electron transfer mechanisms, respectively.

Mechanism	Position	EA		EA^−^	
		HO^•^	Cl_3_COO^•^	HO^•^	Cl_3_COO^•^
HAT	1a	−144.0	−36.3		
	2a	−144.2	−36.4	−167.5	−59.7
	1a’			−150.7	−42.9
	2a’			−151.8	−44.0
RAF	1	−62.6	32.8	−16.9	/
	2	−47.4	39.7	−66.4	8.5
	3	−47.9	32.9	−43.4	33.7
	4	−7.6	78.3	−24.5	49.7
	5	−11.2	66.1	0.5	66.7
	6	−39.9	45.9	−66.8	10.8
	1′			−61.8	31.0
	2′			−47.8	42.0
	3′			−45.9	48.0
	4′			−6.0	75.1
	5′			−6.1	65.8
	6′			−40.0	50.8
SPLET	1a	−161.6		−145.1	
	/	17.6	0.7	−5.6	−22.5
SET	/	127.6	110.72	17.6	0.7

**Table 2:** Activation energies $\Delta G_{a}^{‡}$ (kJ mol^−1^) and rate constants *k* (M^−1^ s^−1^) for exergonic reaction pathways of the reactions of EA and EA^−^ with HO^•^ and CCl_3_OO^•^.

Mechanism	Position	HO^•^				Cl_3_COO^•^			
		EA		EA^−^		EA		EA^−^	
		$\Delta G_{a}^{‡}$	*k*	$\Delta G_{a}^{‡}$	*k*	$\Delta G_{a}^{‡}$	*k*	$\Delta G_{a}^{‡}$	*k*
HAT	1a	~0.0	1.91 × 10^9^			64.9	7.74 × 10^3^		
	2a	~0.0	1.91 × 10^9^	~0.0	1.91 × 10^9^	56.7	7.54 × 10^4^	/	/
	1a’	~0.0	1.91 × 10^9^	~0.0	1.91 × 10^9^	64.9	7.74 × 10^3^	/	/
	2a’	~0.0	1.91 × 10^9^	~0.0	1.91 × 10^9^	56.7	7.54 × 10^4^	198.8	4.06 × 10^−20^
RAF	1	36.9	6.17 × 10^7^	17.0	5.30 × 10^7^				
	2	40.8	1.33 × 10^7^	~0.0	1.91 × 10^9^				
	3	39.3	2.47 × 10^7^	26.8	4.60 × 10^7^				
	4	46.5	1.40 × 10^6^	9.3	3.59 × 10^7^				
	5	52.1	1.58 × 10^7^	49.3	4.12 × 10^5^				
	6	40.7	1.43 × 10^7^	~0.0	1.91 × 10^9^				
	1′	36.9	6.17 × 10^7^	33.7	8.27 × 10^7^				
	2′	40.8	1.33 × 10^7^	37.4	4.69 × 10^7^				
	3′	39.3	2.47 × 10^7^	36.2	7.02 × 10^7^				
	4′	46.5	1.40 × 10^6^	44.3	3.12 × 10^6^				
	5′	52.1	1.58 × 10^5^	51.9	1.49 × 10^5^				
	6′	40.7	1.43 × 10^7^	38.4	3.14 × 10^7^				
SPLET(I)	1a	/	/	/	/	~0.0	1.91 × 10^9^	/	/
SPLET(II)	/	/	/	/	/	0.7	1.56 × 10^9^	/	/
SET	/	/	/	/	/	/	/	/	/
$k_{overall}$		9.70 × 10^9^				1.59 × 10^9^			
$k_{overall}^{exp}$		8.9 × 10^9^				0.84 × 10^9^			

## Supplementary Materials

The supplementary materials are available online at https://www.mdpi.com/2076-3921/9/7/587/s1.
