# Supplementary material for: Antioxidative Action of Ellagic Acid—A Kinetic DFT Study
Source: Antioxidants (Basel). 2020 Jul 6;9(7):587. doi: 10.3390/antiox9070587 (PMC7402119; doi:10.3390/antiox9070587)
Supplement: Supplementary file 1 [file antioxidants-09-00587-s001.pdf]

Antioxidants

Supplementary Materials

Antioxidative Action of Ellagic Acid – A Kinetic DFT Study

Jelena Tošović[1,2] • Urban Bren[1,3,*]

[1]*University of Maribor, Faculty of Chemistry and Chemical Technology, Smetanova street 17, SI-2000 Maribor, Slovenia*

[2]*University of Kragujevac, Faculty of Science, Department of Chemistry, 12 Radoja Domanovića, 34000 Kragujevac, Serbia*

[3]*University of Primorska, Faculty of Mathematics, Natural Sciences and Information Technologies, Glagoljaška 8, SI-6000 Koper, Slovenia*

[*] Corresponding author
E-mail address: urban.bren@um.si (U.Bren)

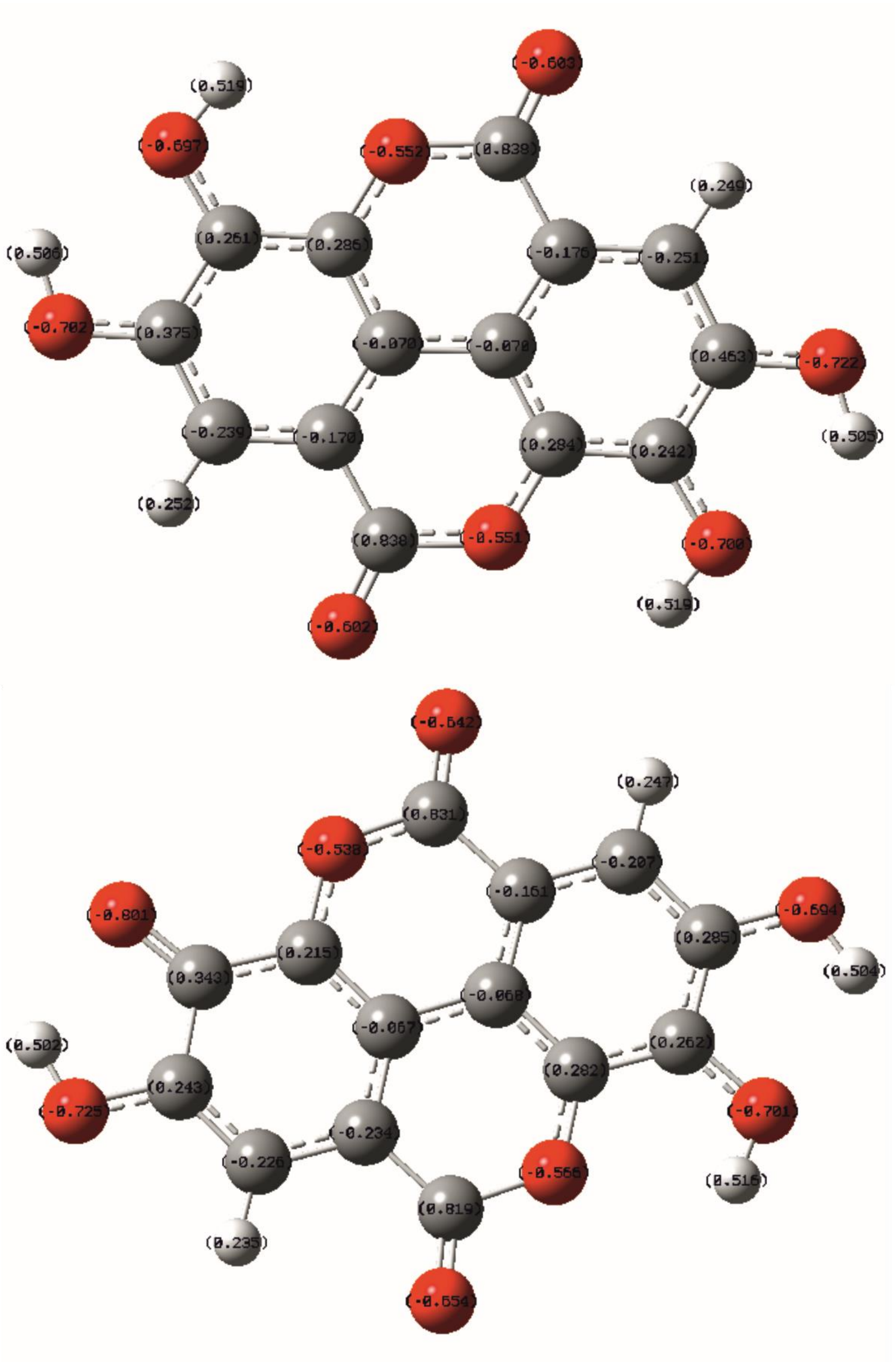

Figure S1. NBO charge distribution in ellagic acid (top) and its monoanion (bottom).

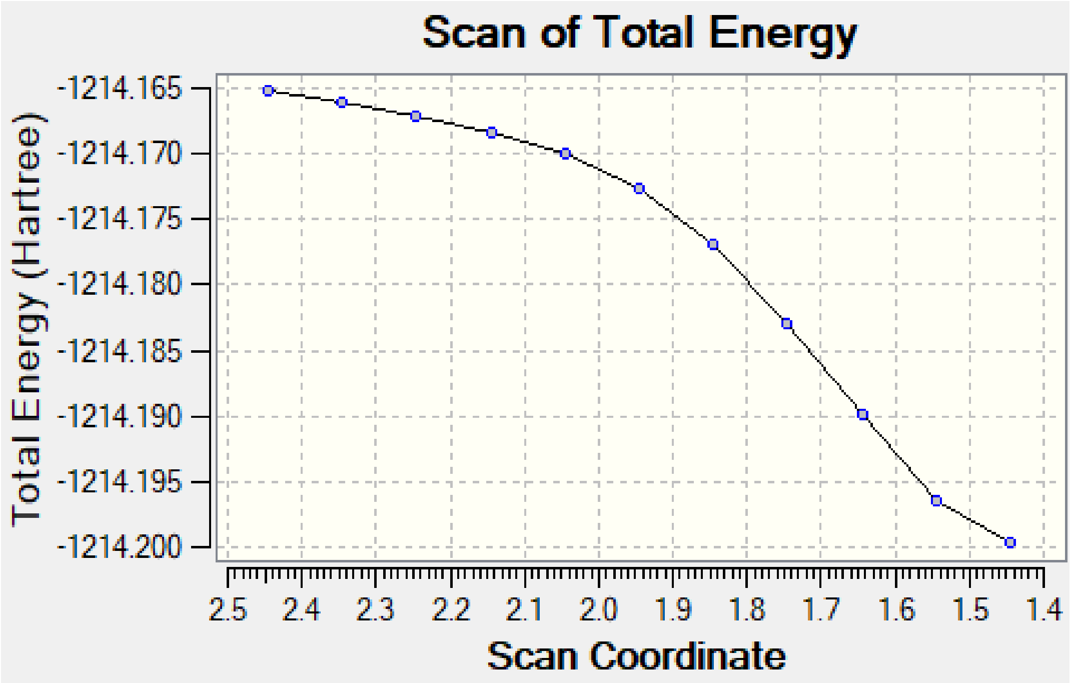

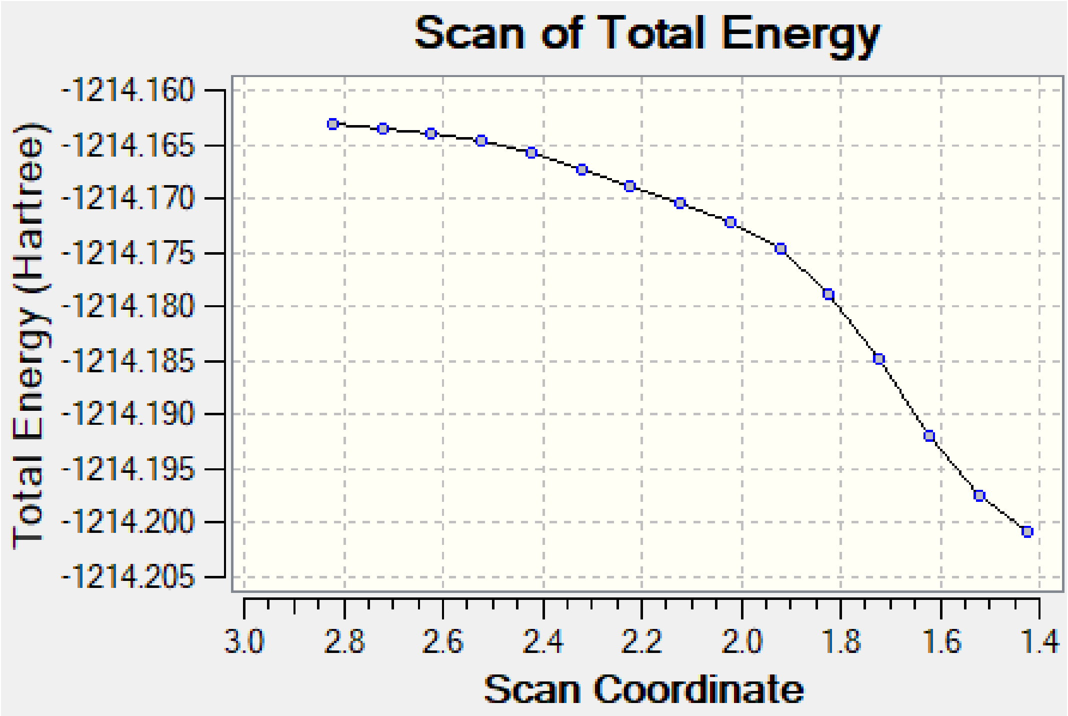

Figure S2. Energy profiles for RAF pathways of **EA**$^{-}$ reacting with HO$^{\bullet}$ at positions 2 (top) and 6 (bottom). The scan coordinates represent the corresponding HO$^{\bullet}$–C2 and HO$^{\bullet}$–C6 distances in Å.

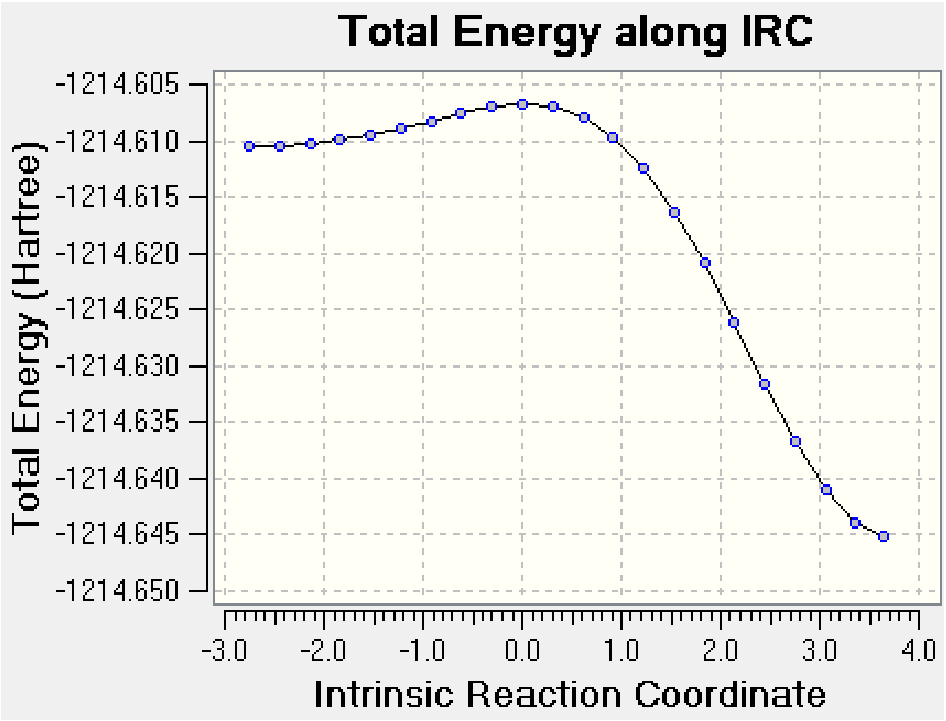

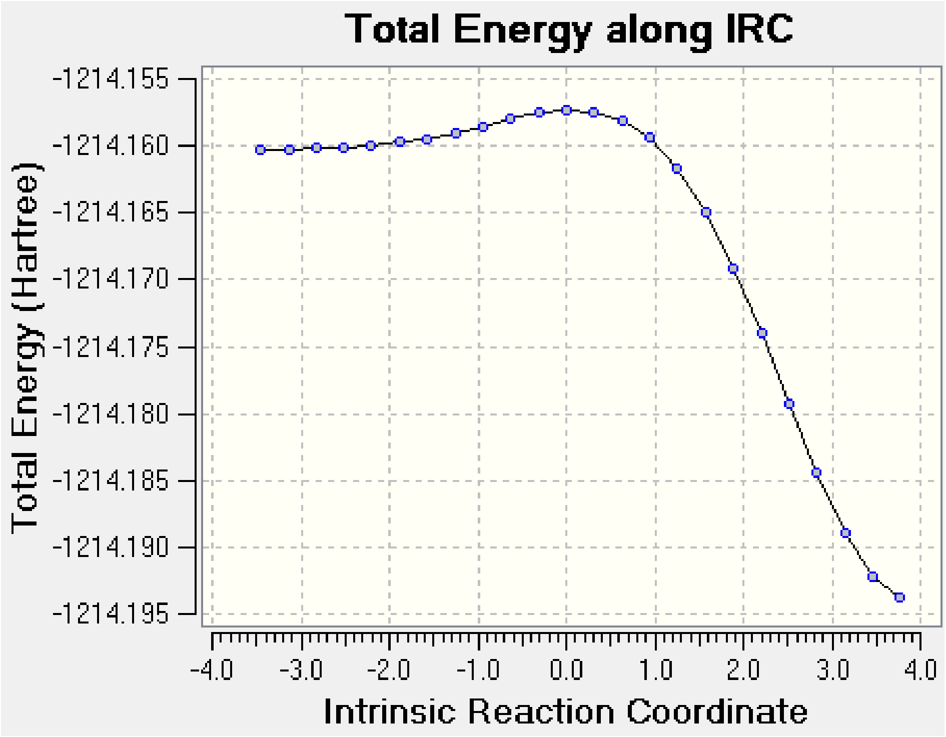

Figure S3. Results of the representative IRC calculations for the TSs of the RAF reaction of $HO^{\bullet}$ with ellagic acid at position 1 (top) and its monoanion at position 1a'(bottom).

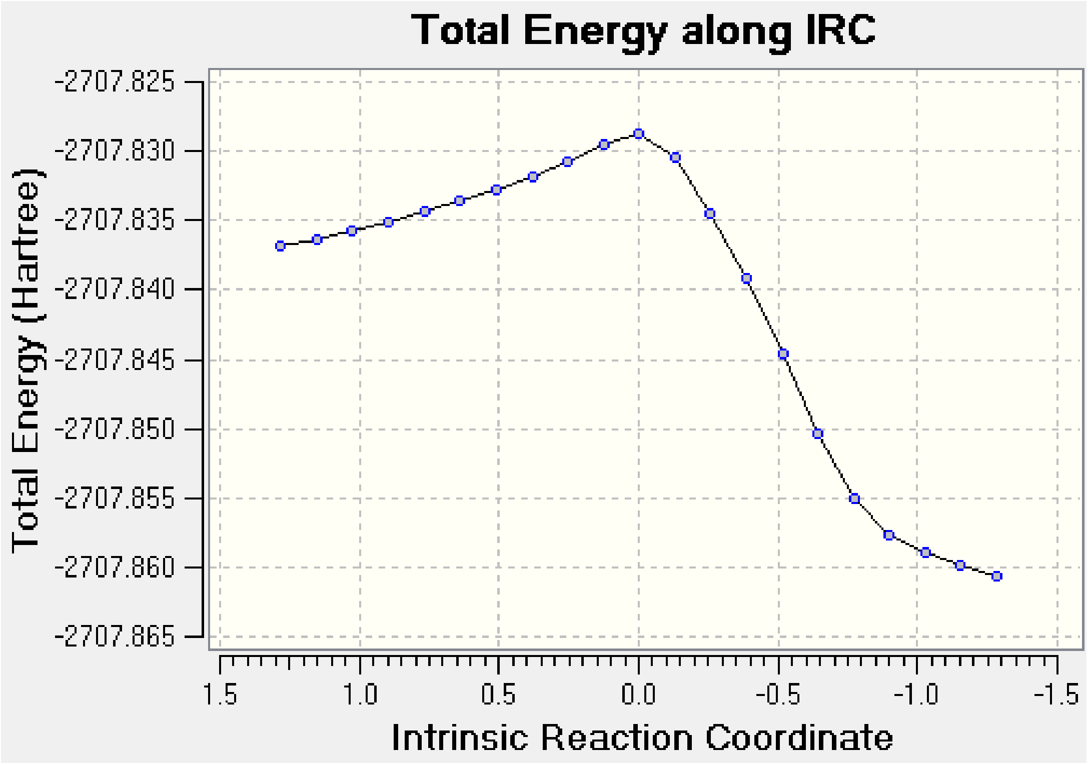

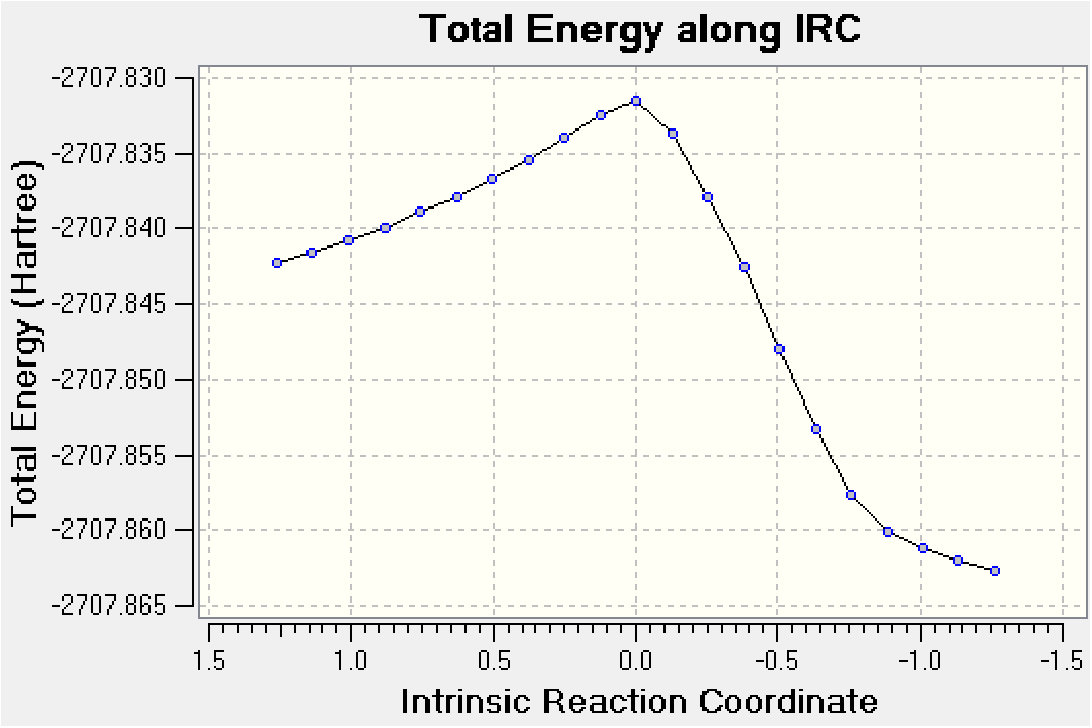

Figure S4. Results of the IRC calculations for the TSs of the HAT reaction of $CCl_3OO^{\bullet}$ with ellagic acid at positions 1a (top) and 2a (bottom).

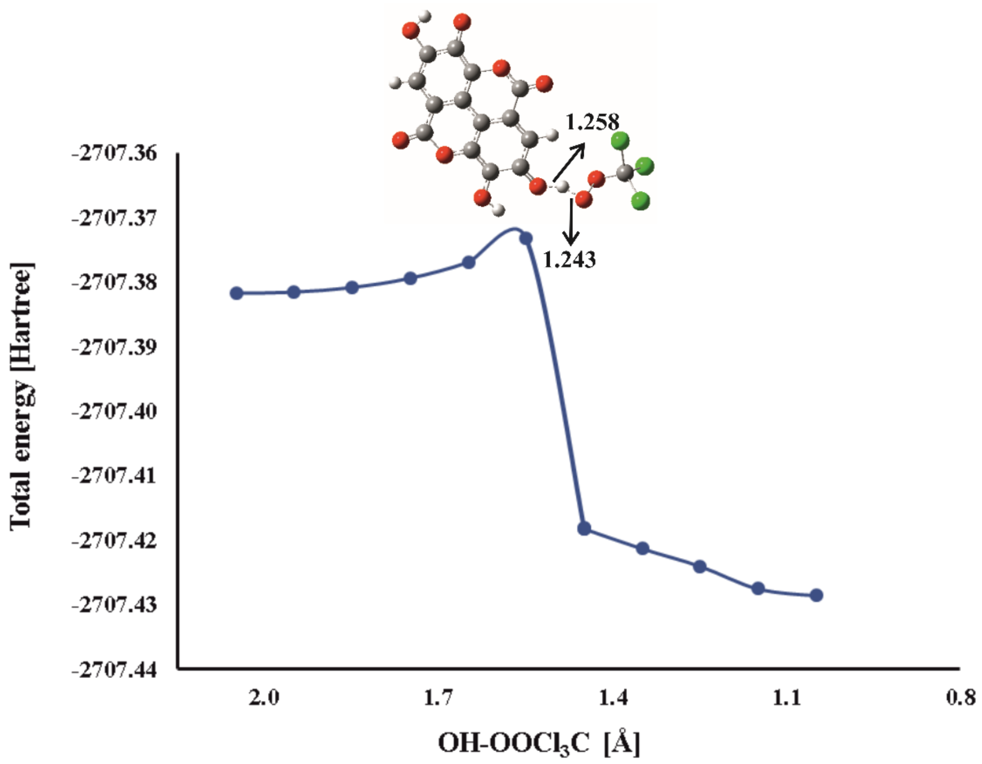

Figure S5. Dependence of total energy on the OH–•OOCl3C distance for the HAT pathway of ellagic acid monoanion at position 2a'. All distances are reported in Å.

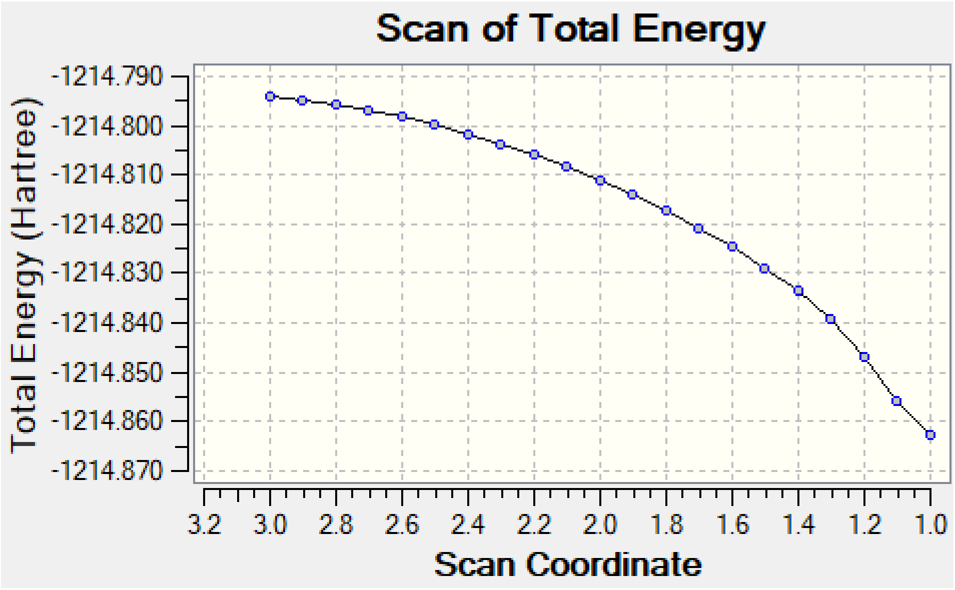

Figure S6. Dependence of total energy on the HO–H distance (Å) in the proton transfer reaction from ellagic acid at position 1a to HO^-.

Table S1. Branching ratios (Γ) in % for all thermodynamically feasible mechanistic pathways of ellagic acid.

Mechanism	Position	HO$^{\bullet}$	
		**EA**	**EA^{-}**
HAT	1a	4.2	/
	2a	4.2	17.6
	1a’	4.2	17.6
	2a’	4.2	17.6
RAF	1	0.1	0.5
	2	0.0	17.6
	3	0.1	0.4
	4	0.0	0.3
	5	0.0	0.0
	6	0.0	17.6
	1’	0.1	0.8
	2’	0.0	0.4
	3’	0.1	0.6
	4’	0.0	0.0
	5’	0.0	0.0
	6’	0.0	0.3
		$CCl_3OO^{\bullet}$	
HAT	2a	0.0	/
	1a’	0.0	/
	2a’	0.0	/
SPLET (I)	1a	55.1	/
SPLET (II)	/	44.9	/

Considering that the predominant form of **Tx** at physiological conditions represents the carboxylate monoanion (**Tx**$^{-}$) (Alberto et al., *PCCP*, 2013) (Figure S7), the following reactions were examined:

HAT: $\mathbf{Tx}^{-} + Cl_3COO^{\bullet} \rightarrow \mathbf{Tx}^{\bullet -} + Cl_3COOH$

RAF: $\mathbf{Tx}^{-} + Cl_3COO^{\bullet} \rightarrow [\mathbf{Tx} - OOCl_3C]^{\bullet -}$

SPLET (I step): $\mathbf{Tx}^{-} + HO^{-} \rightarrow \mathbf{Tx}^{2-} + H_2O$

SPLET(II step): $\mathbf{Tx}^{2-} + Cl_3COO^{\bullet} \rightarrow \mathbf{Tx}^{\bullet -} + Cl_3COO^{-}$

SET: $\mathbf{Tx}^{-} + Cl_3COO^{\bullet} \rightarrow \mathbf{Tx}^{\bullet} + Cl_3COO^{-}$

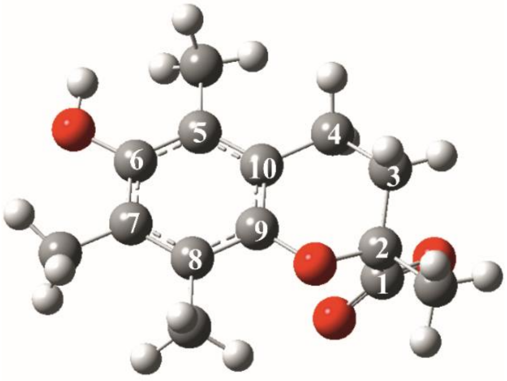

Figure S7. The optimized structure of carboxylate monoanion of trolox (**Tx**$^{-}$). The atom labeling scheme is applied throughout the Supporting Materials.

The Gibbs free energies of the investigated reactions are collected in Table S2. Endergonic radical adduct formation, as well as electron transfer reactions, indicate that RAF and SET mechanism are not operative. Both steps of the SPLET mechanism are characterized with negative ΔG_r values. This finding indicates that HO^{-} is able to uptake a proton from **Tx**$^{-}$ to yield **Tx**$^{2-}$. Analogous to the case of **EA,** considering that **Tx**$^{2-}$ is formed at higher pH values only (pH>11),

it is reasonable to suppose that SPLET mechanism cannot be responsible for the antioxidative activity of **Tx** toward the $CCl_3OO^•$ radical at physiological conditions (Alberto et al., *PCCP*, 2013). Taking all these facts into account, it is apparent that **Tx**$^–$ can readily scavenge $CCl_3OO^•$ radical only via the HAT mechanism. Our numerous attempts to locate TS for the HAT reaction of **Tx**$^–$ with $Cl_3COO^•$ all yielded the structure of products: **Tx**$^{•–}$ and Cl_3COOH, thus indicating that the reaction is barrierless. This assumption was confirmed by scanning the total energy as a function of the O6–H6 distance (Figure S8), so it was assigned the rate constant of a diffusion limited reaction (1.91×10^9 $M^{-1}s^{-1}$).

Table S2. Gibbs free energies ΔG_r (kJ mol^{-1}) of the reactions between **Tx**$^–$ and $CCl_3OO^•$.

Mechanism	HAT	RAF							SPLET (I)	SPLET (II)	SET
Position	6	1	5	6	7	8	9	10	1	/	/
ΔG_r	-72.3	121.5	25.2	12.6	34.0	42.3	42.5	46.4	-53.9	-143.0	6.9

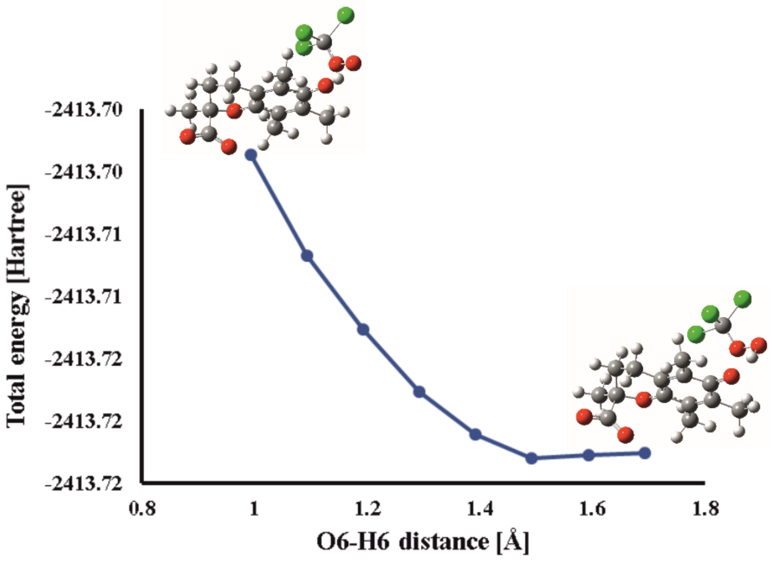

Figure S8. Dependence of total energy on the O6–H6 distance in the hydrogen atom transfer reaction from trolox to $CCl_3OO^•$.

Cartesian coordinates in Å for all transition states revealed for the RAF reaction mechanism of ellagic acid or its monoanion with HO•

TS1(1')

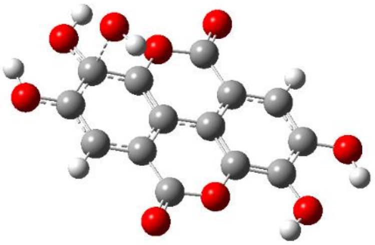

0 2	x	y	z
C	3.24441900	0.72670700	-0.25210300
C	2.34394100	1.78150400	-0.18681700
C	0.98146200	1.50509000	-0.12803100
C	0.52419600	0.18174300	-0.12677300
C	1.41089200	-0.86992200	-0.19908000
C	2.79670400	-0.62705000	-0.20190500
C	-0.01099500	2.59799700	-0.05881200
C	-0.86895300	-0.11710600	-0.07242900
C	-1.76339000	0.93731000	-0.01228200
C	-3.12584200	0.69503100	0.04249200
C	-3.58393000	-0.63704200	0.03669500
C	-2.69219100	-1.69704000	-0.02446300
C	-1.32326900	-1.43484500	-0.08011000
C	-0.34016700	-2.52564900	-0.15501100
H	2.70833700	2.80027600	-0.19921400
H	-3.06641100	-2.71265700	-0.03017800
O	3.71013600	-1.56927000	-0.46383400
H	3.36327600	-2.44844400	-0.25900100
O	4.55712000	0.96678100	-0.33216600
H	5.04236200	0.12950800	-0.35811800
O	0.99991200	-2.16215100	-0.21414700
O	-0.58486000	-3.69680900	-0.17350800
O	-4.91135800	-0.88104400	0.08922000
H	-5.39368800	-0.04477600	0.13400400
O	-4.05935900	1.66290100	0.10063600
H	-3.64963700	2.53829900	0.09707700
O	-1.34053200	2.24063500	-0.00528200
O	0.24925000	3.76665800	-0.04772100
O	3.00977600	-0.73720300	1.90236900
H	2.39781000	-0.08654100	2.28266200

TS2(2’)

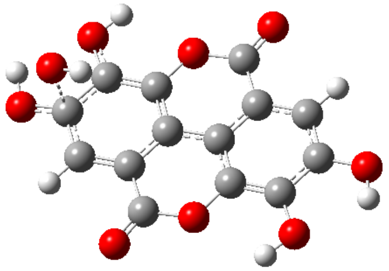

0 2	x	y	z
C	-2.39681000	-1.57739900	-0.25767200
C	-1.02979800	-1.38447800	-0.18063200
C	-0.50737000	-0.08203200	-0.13748900
C	-1.34065900	1.02359300	-0.15888500
C	-2.71212900	0.86059000	-0.24040300
C	-0.10110300	-2.53012100	-0.15944200
C	0.89577700	0.13777900	-0.06072900
C	1.73373500	-0.96645100	-0.03823300
C	3.10639200	-0.79919000	0.03466100
C	3.63423600	0.50525100	0.08162400
C	2.80015100	1.61538900	0.05311100
C	1.42289800	1.43012100	-0.02009900
C	0.50182400	2.57988700	-0.05503400
H	-2.82696700	-2.56927200	-0.31023800
H	3.22824600	2.60895300	0.08773300
O	-3.58689700	1.86427500	-0.30030100
H	-3.14012500	2.72063900	-0.24053200
O	-4.55460600	-0.63851500	-0.51358300
H	-5.04061200	0.17996600	-0.34312500
O	-0.84828300	2.29982100	-0.12463500
O	0.82366300	3.73313800	-0.02837500
O	4.96952500	0.67788800	0.15414500
H	5.41001300	-0.18244900	0.16297500
O	3.98810400	-1.81593900	0.06381000
H	3.53433600	-2.66851300	0.02657700
O	1.24665700	-2.24418800	-0.08463500
O	-0.41874300	-3.68352500	-0.20007500
O	-3.50144100	-0.41499400	1.85733600
H	-2.60664700	-0.42799400	2.23111900
C	-3.25748900	-0.46044300	-0.22811500

TS3(3’)

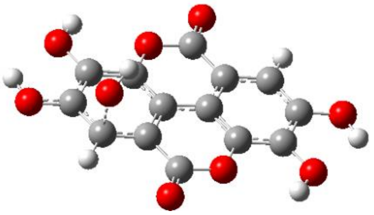

0 2	x	y	z
C	-2.52790100	-1.35065100	-0.25299200
C	-1.12245100	-1.23125000	-0.23806100
C	-0.53767800	0.02006000	-0.13057600
C	-1.31876600	1.17709200	-0.10471800
C	-2.69651400	1.09427500	-0.18046700
C	-0.26953200	-2.42554100	-0.26301400
C	0.87798800	0.16795100	-0.06522100
C	1.65478400	-0.97850700	-0.10045600
C	3.03633000	-0.88736600	-0.03449100
C	3.63220700	0.38281500	0.07106200
C	2.85763000	1.53470000	0.10597800
C	1.47107100	1.42581500	0.03844500
C	0.61192600	2.62039400	0.06850300
H	3.33845300	2.50114400	0.18763100
O	-3.52053400	2.15827400	-0.17826000
H	-3.02079000	2.98331600	-0.11521400
O	-4.62543300	-0.27311300	-0.40751300
H	-5.02781800	0.60657100	-0.37581200
O	-0.75652400	2.41491800	-0.00353100
O	0.98819100	3.75323100	0.15018400
O	4.97666400	0.48265100	0.13890600
H	5.37174200	-0.39873700	0.10464600
O	3.86083000	-1.95108400	-0.06242600
H	3.35980300	-2.77446100	-0.13487600
O	1.09588900	-2.22178400	-0.20209800
O	-0.65516400	-3.55791400	-0.33455500
H	-2.99816200	-2.29497100	-0.48688900
O	-2.96906100	-1.77730800	1.76485500
H	-2.41032000	-1.09844000	2.17553100
C	-3.29772100	-0.17435000	-0.29391400

TS4(4')

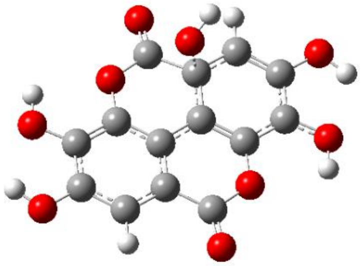

0 2	x	y	z
C	2.65755900	1.68396400	0.02528500
C	1.27848200	1.50062900	-0.01302500
C	0.74972400	0.20935700	-0.08126500
C	1.58577400	-0.89687600	-0.13439900
C	2.95920500	-0.73009700	-0.09783700
C	0.35515100	2.64866000	0.00353400
C	-0.65094400	-0.00128500	-0.09986600
C	-1.48468900	1.09127200	-0.08315600
C	-2.86389200	0.91672400	-0.10054400
C	-3.39810700	-0.40040000	-0.14730600
C	-2.57247100	-1.49987900	-0.15132700
C	-1.17299300	-1.31876200	-0.02697900
C	-0.24475400	-2.45240800	-0.28821600
H	-2.99350600	-2.49547000	-0.20876400
O	3.84249500	-1.74483000	-0.14803800
H	3.38803000	-2.59563500	-0.20935400
O	4.82657600	0.74100400	0.02765400
H	5.26563400	-0.11926400	-0.01283300
O	1.10083200	-2.16999200	-0.26962700
O	-0.57824800	-3.58006000	-0.50011300
O	-4.73515100	-0.55909800	-0.20491800
H	-5.17071700	0.30349400	-0.22022900
O	-3.74192500	1.92321400	-0.09290600
H	-3.29355200	2.78068100	-0.07419700
O	-0.99828100	2.36744600	-0.05743500
O	0.67339900	3.80186200	0.05455400
H	3.08865000	2.67531800	0.08055300
C	3.48959500	0.57257500	-0.01066000
O	-0.74495800	-1.58741800	1.90513000
H	-1.56829100	-2.02699600	2.16497300

TS5(5')

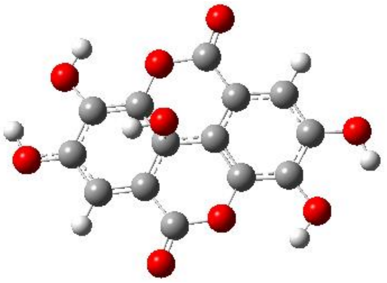

0 2	x	y	z
C	2.59704300	1.66475200	-0.16356200
C	1.22588400	1.43963200	-0.04494100
C	0.73934200	0.13847400	0.01230200
C	1.59984400	-0.94035000	-0.04054300
C	2.96560700	-0.73770500	-0.16169500
C	0.27536800	2.56150300	0.02761500
C	-0.66560200	-0.12132000	0.15882600
C	-1.53704700	0.97061200	-0.02499000
C	-2.88380900	0.76562200	-0.22188200
C	-3.36490200	-0.55839600	-0.31324500
C	-2.50006200	-1.65670800	-0.23073800
C	-1.15160600	-1.43826300	-0.04141200
C	-0.18887700	-2.55640300	-0.00050100
H	-2.90053800	-2.65676100	-0.33726600
O	3.86997700	-1.73316300	-0.22557800
H	3.43713600	-2.59624700	-0.18276500
O	4.78832200	0.78588600	-0.34000000
H	5.24787200	-0.06381000	-0.36519700
O	1.14904600	-2.23353000	0.02579100
O	-0.47980200	-3.71791900	0.00158100
O	-4.67134300	-0.77234100	-0.50191800
H	-5.14144800	0.07234800	-0.55417700
O	-3.79573800	1.74874300	-0.35604600
H	-3.38066000	2.61885300	-0.28824100
O	-1.07706700	2.24671100	0.03255600
O	0.55963200	3.72208600	0.07438200
H	2.99591100	2.66984600	-0.21191500
C	3.45839900	0.57935600	-0.22148000
O	-0.61538400	-0.08234900	2.18118600
H	-1.49691000	-0.42205500	2.40141300

TS6(6')

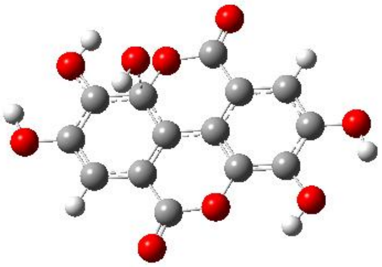

0 2	x	y	z
C	-2.60994700	-1.66303300	-0.08733100
C	-1.24350500	-1.40342000	-0.12668700
C	-0.79025000	-0.08402400	-0.10139900
C	-1.68360400	0.97339300	-0.04620500
C	-3.04748700	0.73184800	-0.01187800
C	-0.25890900	-2.49630800	-0.22601600
C	0.59598300	0.21635700	-0.11909800
C	1.49500400	-0.86404600	-0.09094300
C	2.87420800	-0.60927100	-0.20052100
C	3.33353800	0.71968100	-0.16827200
C	2.43784300	1.77509800	-0.11298300
C	1.05904000	1.52146300	-0.08488800
C	0.08079800	2.62147800	-0.04355200
H	2.81197600	2.79087400	-0.09061000
O	-3.98228400	1.69938800	0.04221200
H	-3.57262900	2.57474300	0.05012100
O	-4.82873300	-0.84649100	0.00547600
H	-5.31426500	-0.01186700	0.05087100
O	-1.25560200	2.27094100	-0.01951900
O	0.34420300	3.78975600	-0.02548700
O	4.66084600	0.96849900	-0.21051000
H	5.15007600	0.13596800	-0.24419800
O	3.78383100	-1.58111400	-0.27631800
H	3.36164900	-2.45221500	-0.28897500
O	1.07409000	-2.13687000	-0.32867900
O	-0.51523700	-3.66408700	-0.27152400
H	-2.98757200	-2.67724500	-0.10746000
C	-3.50307500	-0.59948300	-0.02952100
O	1.51981400	-1.14176800	1.95880600
H	1.70552000	-0.26467400	2.33036400

TS1

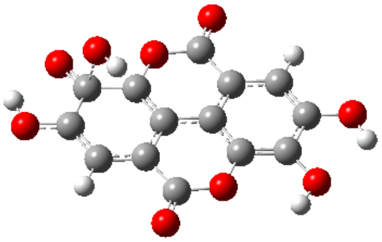

-1 2	x	y	z
C	3.28901200	0.64168200	-0.22133500
C	2.41974200	1.71179300	-0.18142300
C	1.04373600	1.45854000	-0.14148700
C	0.56631300	0.12690600	-0.14920100
C	1.41790400	-0.94504700	-0.19992700
C	2.85832800	-0.77069500	-0.23665900
C	0.08757100	2.56231000	-0.08987100
C	-0.83839000	-0.12801600	-0.08857900
C	-1.70840500	0.94985200	-0.03408000
C	-3.07543000	0.74322100	0.03389200
C	-3.57347100	-0.57365300	0.04582900
C	-2.71183300	-1.65624600	-0.01069800
C	-1.33550700	-1.43053800	-0.07849600
C	-0.38045100	-2.54528400	-0.14397200
H	2.79712700	2.72629100	-0.18630600
H	-3.11015600	-2.66263500	-0.00211000
O	3.69103400	-1.65821500	-0.48046900
O	4.60933600	0.81115100	-0.29051800
H	4.98521700	-0.08651700	-0.35957500
O	0.95284400	-2.22430600	-0.21146700
O	-0.67035200	-3.71319000	-0.14772600
O	-4.91004900	-0.77744300	0.11332500
H	-5.36435400	0.07450000	0.14761900
O	-3.98016800	1.74216000	0.09054400
H	-3.53815900	2.60147000	0.07633700
O	-1.26032300	2.24269800	-0.04120700
O	0.36160400	3.73422300	-0.08699300
O	2.89989600	-0.61011500	1.93644200
H	2.32502500	0.10224600	2.24464100

TS1’

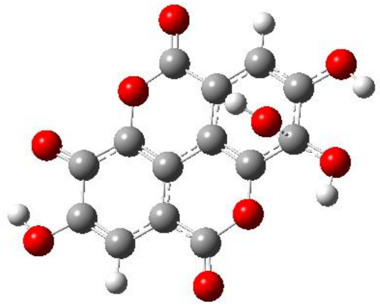

-1 2	x	y	z
C	3.23604800	0.68930300	-0.25143900
C	2.35376200	1.75678800	-0.18937000
C	0.98500300	1.50187900	-0.13458100
C	0.49816100	0.19049800	-0.13490400
C	1.37323600	-0.87607500	-0.20631700
C	2.76104600	-0.65218300	-0.20824200
C	0.00773800	2.61004900	-0.06664200
C	-0.90459900	-0.08313400	-0.08114700
C	-1.77878000	0.98235600	-0.01756600
C	-3.18457900	0.80114500	0.04968900
C	-3.60639500	-0.58857100	0.04125800
C	-2.74565600	-1.65246800	-0.02473800
C	-1.35570900	-1.41279900	-0.08589500
C	-0.40988300	-2.49985900	-0.16219100
H	2.73169500	2.77058100	-0.19676800
H	-3.12833800	-2.66594500	-0.02914300
O	3.65877300	-1.61881000	-0.44738500
H	3.27502700	-2.48661800	-0.26066200
O	4.55779400	0.90425100	-0.32214200
H	5.02369000	0.05643400	-0.33948200
O	0.95262900	-2.15910200	-0.22510100
O	-0.64698100	-3.68189100	-0.18201400
O	-4.95117600	-0.76360700	0.10488100
H	-5.31526800	0.13874400	0.14320600
O	-4.05629600	1.71042500	0.11447600
O	-1.30594900	2.28471500	-0.01413100
O	0.31024200	3.77759800	-0.05760800
O	2.94233400	-0.74768300	1.93411500
H	2.34589300	-0.06194700	2.27464300

TS2’

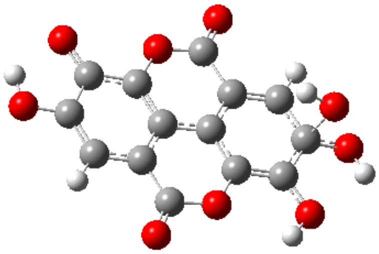

-1 2	x	y	z
C	-2.39553600	-1.56347600	-0.25950100
C	-1.02609000	-1.38728500	-0.18880700
C	-0.47919900	-0.09416500	-0.14857200
C	-1.30327600	1.02136100	-0.16922400
C	-2.67695900	0.87228400	-0.24377600
C	-0.10886700	-2.54399100	-0.16754600
C	0.93076100	0.10272300	-0.06990000
C	1.75231000	-1.00957900	-0.04259800
C	3.16320700	-0.90075800	0.04236800
C	3.65261600	0.46437300	0.08715500
C	2.84600400	1.57474800	0.05279700
C	1.45100700	1.40813300	-0.02833100
C	0.56154400	2.55001000	-0.06423000
H	-2.83555400	-2.55130000	-0.30636400
H	3.27906700	2.56712900	0.08880200
O	-3.53575500	1.89496800	-0.29766900
H	-3.06504900	2.73812500	-0.23623100
O	-4.54744700	-0.59690700	-0.49531600
H	-5.01576000	0.22938700	-0.31492700
O	-0.80660700	2.29112500	-0.13625200
O	0.87519700	3.71468200	-0.03878200
O	5.00097000	0.57084000	0.16870800
H	5.31993200	-0.34979700	0.17730900
O	3.99072500	-1.85337400	0.08034900
O	1.22005200	-2.28667600	-0.09228600
O	-0.46603700	-3.69477100	-0.21051900
O	-3.46631100	-0.36140400	1.88957500
H	-2.56137900	-0.40428000	2.23498500
C	-3.24078300	-0.43723500	-0.23084100

TS3

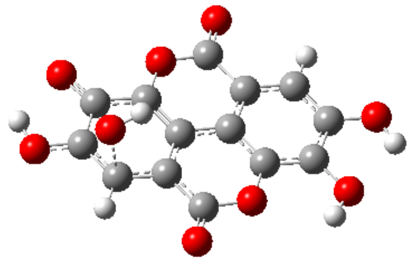

-1 2	x	y	z
C	-2.54088700	-1.36402400	-0.23971300
C	-1.13418200	-1.24768700	-0.22627500
C	-0.56515100	0.03692400	-0.12218300
C	-1.32454100	1.18968800	-0.10591200
C	-2.73931900	1.15337500	-0.20391400
C	-0.30186000	-2.42391300	-0.22030900
C	0.85739500	0.16742900	-0.05734700
C	1.63141000	-0.98478300	-0.08574400
C	3.01351300	-0.89789800	-0.02874900
C	3.62117100	0.36576000	0.06153000
C	2.85402100	1.52167600	0.09163300
C	1.46621300	1.41950300	0.03263500
C	0.61242900	2.62071400	0.05779100
H	3.33818500	2.48726400	0.16220000
O	-3.52454100	2.13111000	-0.22681400
O	-4.61932400	-0.23599100	-0.48571500
H	-4.91773000	0.69312000	-0.44518200
O	-0.73085400	2.43461700	-0.00872600
O	1.02549100	3.75139700	0.13372700
O	4.96973900	0.45413800	0.11972800
H	5.35324400	-0.43217000	0.08803100
O	3.82781200	-1.97459600	-0.05246100
H	3.30872500	-2.78736400	-0.11740700
O	1.08031700	-2.22881200	-0.17085100
O	-0.66719700	-3.57427900	-0.25974100
H	-3.01229000	-2.32066400	-0.41551300
O	-3.18981400	-1.35563100	1.80054800
H	-2.33372500	-1.05272700	2.13401000
C	-3.30178800	-0.19061000	-0.32072700

TS3’

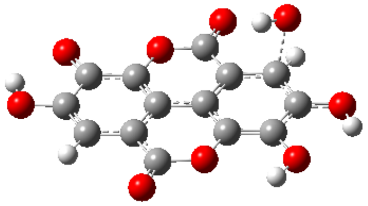

-1 2	x	y	z
C	-2.52535400	-1.33154700	-0.25875300
C	-1.11873400	-1.23251100	-0.23938600
C	-0.50892600	0.00706400	-0.13059900
C	-1.27860000	1.17545000	-0.10726300
C	-2.65797100	1.10894900	-0.18523400
C	-0.27899100	-2.43782100	-0.26622100
C	0.91288200	0.13127100	-0.06548100
C	1.67078900	-1.02335800	-0.10101500
C	3.08932900	-0.99462800	-0.03754800
C	3.65285600	0.33687800	0.07172400
C	2.90737500	1.48758600	0.10908200
C	1.50212200	1.40150000	0.04174600
C	0.67722100	2.58671100	0.06912000
H	3.39463700	2.45181600	0.19176300
O	-3.46260700	2.19267800	-0.18217200
H	-2.93900200	3.00224800	-0.11348600
O	-4.61159600	-0.22470100	-0.40964500
H	-4.99674500	0.66206500	-0.37304100
O	-0.71082600	2.40418200	-0.00610800
O	1.04425900	3.73195800	0.15062800
O	5.00839300	0.36666700	0.13487400
H	5.27702000	-0.56813300	0.08479600
O	3.86102100	-1.99233300	-0.06627400
O	1.06621000	-2.26189800	-0.20375900
O	-0.70359100	-3.56512500	-0.34226800
H	-3.00737500	-2.27239800	-0.48193100
O	-2.99326500	-1.71833900	1.78811900
H	-2.30132300	-1.15472100	2.16805500
C	-3.27919700	-0.14647700	-0.29739300

TS4

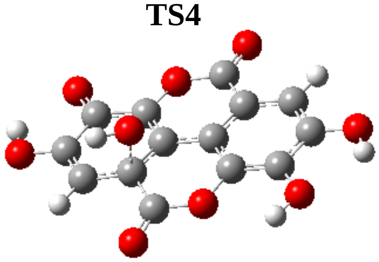

-1 2	x	y	z
C	2.67368200	1.65399200	0.02987800
C	1.28704700	1.49732900	-0.00212400
C	0.72482900	0.22231900	-0.07050100
C	1.54263500	-0.89772100	-0.13710200
C	2.91860700	-0.75794600	-0.10584100
C	0.38601400	2.65927500	0.01562500
C	-0.68769400	0.04440300	-0.08296700
C	-1.49227100	1.14837800	-0.06921600
C	-2.91936300	1.03667200	-0.09824400
C	-3.42431000	-0.33683000	-0.14981100
C	-2.62499300	-1.44207800	-0.14382000
C	-1.21767500	-1.27863800	0.00745300
C	-0.31657600	-2.41478900	-0.28474700
H	-3.05249800	-2.43526100	-0.20257100
O	3.77632900	-1.79826200	-0.16954900
H	3.29172900	-2.63166300	-0.23704500
O	4.82821200	0.66538700	0.01739500
H	5.24106200	-0.20677100	-0.03073900
O	1.03975300	-2.16188600	-0.28895500
O	-0.66232100	-3.54040400	-0.51734300
O	-4.76695900	-0.43634000	-0.22980700
H	-5.10065900	0.47738900	-0.22745700
O	-3.73006200	1.99052400	-0.10374600
O	-0.95791800	2.41281500	-0.04827000
O	0.74041000	3.80969200	0.06836500
H	3.12119700	2.63808000	0.08574900
C	3.48155000	0.53006000	-0.01413500
O	-0.76295700	-1.64546800	1.91161000
H	-1.52561000	-2.19679400	2.12777500

TS4’

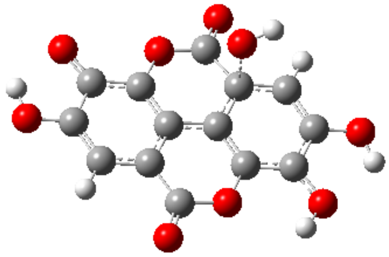

-1 2	x	y	z
C	2.71288000	1.63519100	0.02869600
C	1.31587500	1.47595000	-0.01041200
C	0.78864700	0.17348300	-0.08580400
C	1.60412600	-0.94358300	-0.14287100
C	3.01627000	-0.84103000	-0.10683300
C	0.42866600	2.62027200	0.00760200
C	-0.61911200	-0.00845400	-0.10062900
C	-1.43977300	1.09843600	-0.08242700
C	-2.82036800	0.94465700	-0.10198400
C	-3.38089600	-0.35944500	-0.15338200
C	-2.57447100	-1.47159200	-0.15769000
C	-1.17178800	-1.31327100	-0.03019500
C	-0.26113700	-2.46178500	-0.28907100
H	-3.00940400	-2.46099700	-0.21805100
O	3.84373600	-1.79395900	-0.15435000
O	4.86350300	0.61628400	0.02451200
H	5.17577100	-0.30557700	-0.02866500
O	1.06730400	-2.21287500	-0.28343200
O	-0.63924100	-3.58622600	-0.49198000
O	-4.72386700	-0.49161300	-0.21500800
H	-5.13882000	0.38080200	-0.22899100
O	-3.67855900	1.97316500	-0.09363000
O	-0.94314300	2.36627900	-0.05491700
O	0.74472400	3.78318600	0.06011300
H	3.15318900	2.62327800	0.08826200
C	3.51342000	0.51981900	-0.01138400
O	-0.78826100	-1.58455300	1.92421500
H	-1.62062800	-2.02298200	2.15368700
H	-3.20372000	2.81608100	-0.07213200

TS5

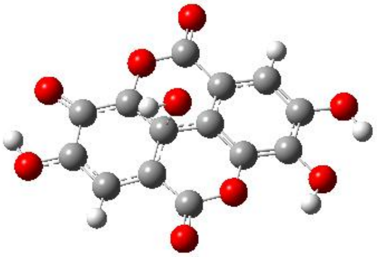

-1 2	x	y	z
C	2.59846700	1.64766500	-0.16113100
C	1.22300000	1.44881300	-0.03588600
C	0.70544300	0.16026900	0.01948500
C	1.54929900	-0.93394000	-0.04316900
C	2.91707400	-0.75702100	-0.17254500
C	0.29284600	2.59085500	0.05953800
C	-0.71029200	-0.06910700	0.17300000
C	-1.55277300	1.03756700	-0.01077800
C	-2.92883500	0.90189400	-0.21368000
C	-3.38555400	-0.48696500	-0.32951700
C	-2.55749400	-1.59870600	-0.26695700
C	-1.20091400	-1.39673100	-0.06289000
C	-0.26415400	-2.51722100	-0.01727000
H	-2.96551400	-2.59448000	-0.38804100
O	3.79934600	-1.77682600	-0.24473700
H	3.33891300	-2.62543100	-0.20435800
O	4.77455100	0.72591500	-0.35964300
H	5.21272000	-0.13457600	-0.38730300
O	1.08477700	-2.22046900	0.02468900
O	-0.56148200	-3.68316600	-0.02871200
O	-4.69738300	-0.60794500	-0.52896500
H	-5.02607000	0.31554200	-0.54937700
O	-3.79824100	1.81243000	-0.32960400
O	-1.03527000	2.31806300	0.08598000
O	0.63666400	3.74598800	0.11489300
H	3.01375100	2.64632100	-0.20481900
C	3.43802900	0.54753500	-0.23186800
O	-0.55913900	-0.36804500	2.15774000
H	-1.46925000	-0.62438000	2.36277800

TS5’

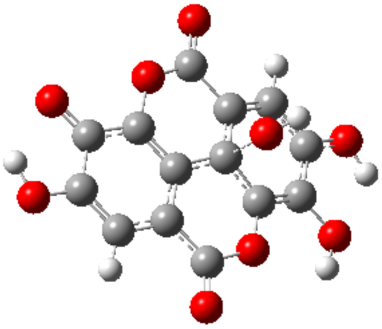

-1 2	x	y	z
C	-2.65372000	-1.62040200	-0.15767200
C	-1.26183300	-1.41877300	-0.03911900
C	-0.77720200	-0.10582500	0.01512400
C	-1.61726600	0.98396500	-0.04245800
C	-3.02491400	0.84329100	-0.16611300
C	-0.34914600	-2.53612300	0.02801900
C	0.63647500	0.12827900	0.15732200
C	1.49479200	-0.97896200	-0.01872600
C	2.84556600	-0.79300700	-0.21869000
C	3.34955200	0.51776400	-0.32024300
C	2.50209100	1.63002200	-0.24213300
C	1.15098300	1.43419800	-0.05039000
C	0.20372400	2.56696100	-0.00585800
H	2.91643800	2.62391100	-0.35345800
O	-3.86856900	1.77942100	-0.23199900
O	-4.82983900	-0.66873500	-0.33890700
H	-5.16823000	0.24396700	-0.36240400
O	-1.11767700	2.27609600	0.02488200
O	0.53726800	3.72579600	-0.00227800
O	4.66237500	0.70885300	-0.51404300
H	5.11482900	-0.14532200	-0.56030700
O	3.73762100	-1.80045900	-0.34614100
H	3.29422800	-2.65604300	-0.27467700
O	1.02616900	-2.24475000	0.04630800
O	-0.62487600	-3.70832200	0.06077200
H	-3.06089900	-2.62327500	-0.20509100
C	-3.48401800	-0.53205900	-0.21802300
O	0.63797300	0.10718000	2.18398700
H	1.52957700	0.44014100	2.36905200

TS6’

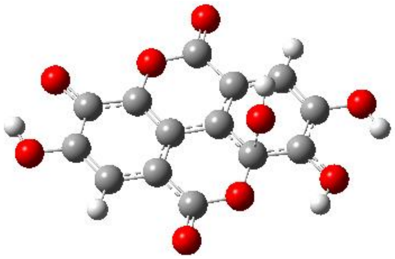

-1 2	x	y	z
C	-2.66842000	-1.62019800	-0.09175000
C	-1.28118600	-1.38413300	-0.12928600
C	-0.82947000	-0.05440300	-0.09727200
C	-1.70335100	1.01558200	-0.04372100
C	-3.11030200	0.83537700	-0.00478900
C	-0.33350100	-2.47395200	-0.22218100
C	0.56572200	0.22040700	-0.11645700
C	1.45291200	-0.87226200	-0.09376600
C	2.83552400	-0.63730100	-0.20688700
C	3.31904500	0.68070200	-0.18156500
C	2.43970500	1.74877100	-0.12288600
C	1.05719000	1.51626500	-0.08682000
C	0.09413100	2.63048900	-0.04467200
H	2.82682500	2.75975600	-0.10377600
O	-3.98356800	1.74511000	0.05119900
O	-4.87411200	-0.72943300	0.00548300
H	-5.24015600	0.17249600	0.04285300
O	-1.22616600	2.31196200	-0.01657700
O	0.39906100	3.79778300	-0.02930300
O	4.65370500	0.90628600	-0.23108300
H	5.12453300	0.06339700	-0.25962100
O	3.72641400	-1.63055900	-0.28380700
H	3.27853000	-2.48866500	-0.28771000
O	1.02297900	-2.13902800	-0.30773700
O	-0.58248200	-3.65212300	-0.27015000
H	-3.05519800	-2.63190100	-0.11533900
C	-3.53007500	-0.55231200	-0.03180100
O	1.55159500	-1.10779500	1.98808800
H	1.71849500	-0.21091600	2.31781700
